# Changes in work status after cancer diagnosis and their associations with depressive symptoms among cancer survivors: findings from the Korean longitudinal study of ageing

**DOI:** 10.1186/s40359-024-01970-9

**Published:** 2024-10-14

**Authors:** Da-eun Lee, Yeonjin Kim, Young-mee Kim, Sung-il Cho

**Affiliations:** 1 Department of Public Health Sciences, Graduate School of Public Health, Seoul National University, 1 Gwanak-ro, Gwanak-gu, Seoul, 08826 Republic of Korea; 2 Institute of Health and Environment, Seoul National University, 1 Gwanak-ro, Gwanak-gu, Seoul, 08826 Republic of Korea

**Keywords:** Depression, Job Security, Mental Health, Resilience, Return to work, Unemployment, Cohort study, Cancer survivors, Survivorship

## Abstract

**Purpose:**

Cancer survivors encounter significant psychological suffering and are prone to develop depressive symptoms. Work contributes to personal fulfillment and social connections, and by doing so, enhances a cancer survivor’s resilience against adversities. However, maintaining employment can be challenging for some cancer survivors. This research aimed to identify the association between changes in work status and depressive symptoms among cancer survivors in South Korea.

**Methods:**

This study used the panel data from the Korean Longitudinal Study of Ageing (KLoSA) and included 199 cancer survivors–799 observations–aged 65 or younger, between 2005 and 2018. Changes in work status consisted of continuous unemployment or employment, quitting a job, and getting a job. We defined depressive symptoms as a CES-D-10 score of 10 or higher and a cut-off of 4 was utilized for sensitivity analysis. Multivariable generalized estimating equation was employed to estimate the odds ratio (OR), adjusting for the number of household members, education level, sex, age, marital status, occupations, cancer treatment, cancer type, catastrophic healthcare expenditure (CHE), and survivorship. Subgroup analysis and interaction between changes in work and cancer types were further explored.

**Results:**

For depressive symptoms, the OR of the continuous unemployment group was 2.27 (95% CI = 1.10–4.69), and the OR of the group that quit a job was 2.20 (95% CI = 1.03–4.72), compared to the continuous employment group. As survivorship increased, the odds of depressive symptoms decreased (OR = 0.94, 95% CI = 0.89–1.00). CHE was associated with depressive symptoms (OR = 2.23, 95% CI = 1.18–4.20). In cancer types with a low tendency to depression, continuous unemployment was associated with depressive symptoms (OR = 3.19, 95% CI = 1.12–9.06). In sensitivity analysis, changes in work, survivorship, and CHE were consistently associated with depressive symptoms.

**Conclusions:**

Cancer survivors who quit a job or continued unemployment were more likely to experience depressive symptoms. The findings of this study imply that assistance for cancer survivors to continue a job or return to employment, including adjustment of workload and hours, may be helpful. Psychological care may be crucial, particularly in the early stage of cancer survivorship. Furthermore, support may be needed to alleviate the burden of healthcare expenditure.

**Supplementary Information:**

The online version contains supplementary material available at 10.1186/s40359-024-01970-9.

## Introduction

Once, cancer was perceived as an incurable disease that could not be survived, but it is no longer considered an insurmountable illness. Worldwide, the 5-year relative survival rate increased from 49% in the mid-1970s to 68% in the mid-2000s [1]. Similarly, in Korea, the 5-year survival rate drastically increased from 42.9% in the mid-1990s to 71.5% in the mid-2000s [2]. Cancer survivors are on the rise and 47% of survivors have been living more than 10 years since diagnosis [3].

In tandem with this trend, the mental health experiences and physical well-being of cancer survivors have drawn growing attention. Cancer survivors encounter significant life alterations, necessitating continuous adaptation throughout their treatment journey [4]. The process can serve as a significant source of stress, and it manifests in a myriad of long-term psychological effects, such as intense emotional upheaval, depression, anxiety, disrupted sleep patterns, and chronic fatigue [5, 6]. These accumulated effects increase a cancer survivor’s sense of personal distress, reduce the capacity and desire for effective social function and contributes to premature mortality [7].

To withstand and rise above challenges in the extreme environment of cancer, emotional and cognitive resilience is needed. The American Psychological Association (APA) defines resilience as: “the process and outcome of successfully adapting to difficult or challenging life experiences, especially through mental, emotional and behavioural flexibility, and adjustment to external and internal demands” [8]. Resilience not only mitigates the initial shock of a cancer diagnosis but also provides a foundation to confront and surmount the associated adversities, ultimately steering a cancer survivor toward improved mental well-being and more successful treatment outcomes [9]. Resilience can also be cultivated through work, enabling people to reach self-realization, self-efficacy, and fellowship in the community of the work setting [10]. Accordingly, previous studies found that employment in the general population is associated with a low prevalence of depression [11]. However, regardless of their intention to remain at work, cancer survivors sometimes are required to cease employment due to the frequent treatments, side effects, and severe pain or loss of mobility [12, 13]. There is a considerable amount of research on the prevalence and trajectory of depression in cancer survivors [5, 6, 14]. Yet, the relationship between depression in cancer survivors and employment status has received limited attention.

Given that resigning from a job can be an inevitable choice that induces weak resilience and depression, which makes cancer survivors more vulnerable to suicidal tendencies than the general population [15], research is needed to identify whether quitting a job is related to depression in cancer survivors. There is a lack of research that concurrently investigates depression and employment changes in cancer survivorship. To date, caregivers’ employment changes and depression have been observed [16]. Among employable cancer patients, one study in Germany found an association between depression and employment status one year after hospitalization, a relatively short follow-up period [17].

Therefore, we aimed to investigate the types of changes in work status after cancer diagnosis in the long term, and to identify their association with depressive symptoms among cancer survivors, using longitudinal data across Korea.

## Materials and methods

### Study design & study population

**Fig. 1:**
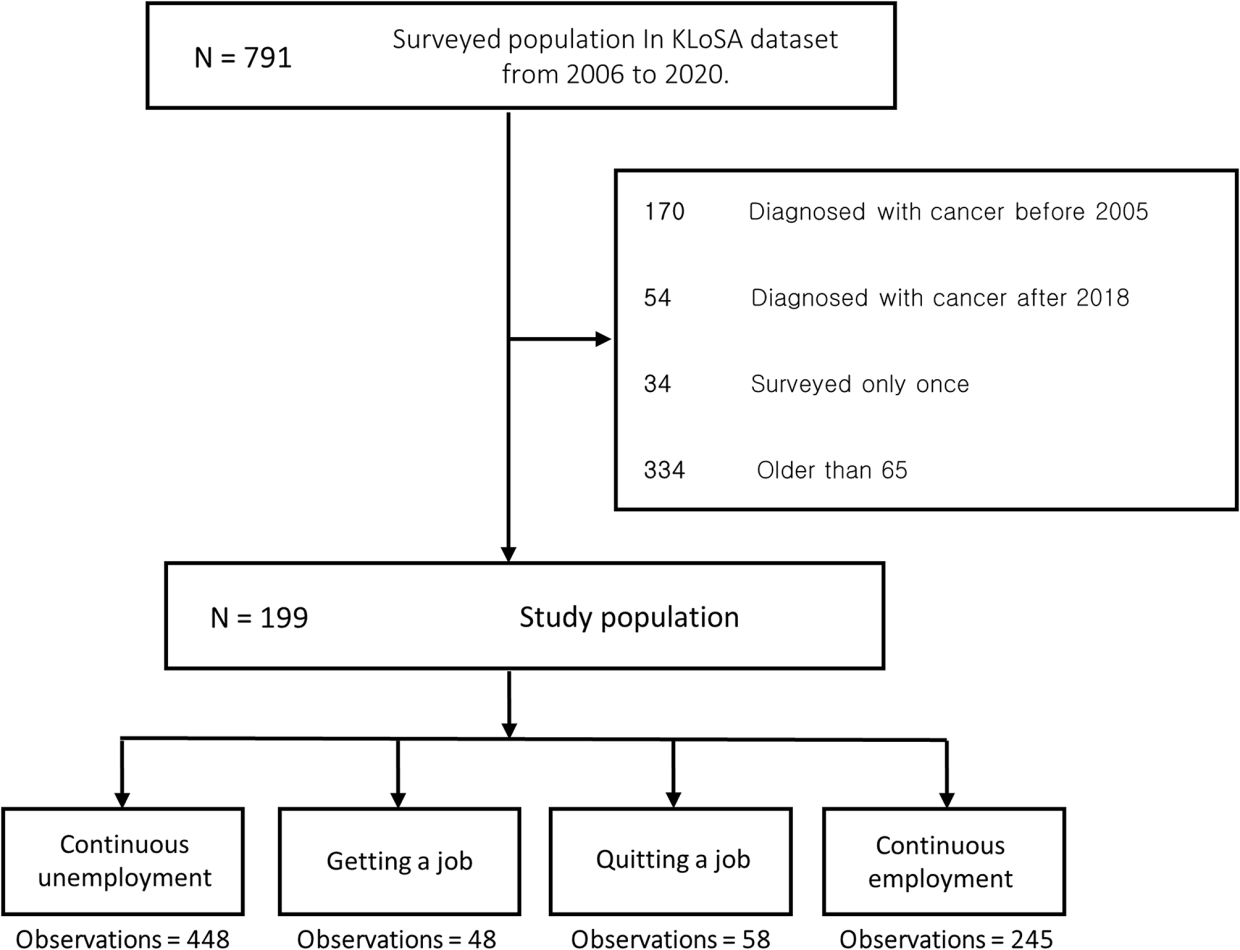
Flow chart detailing the numbers following exclusion criteria and the classification of main groups based on the exposure variable

This is a secondary data analysis, using the panel data from the Korean Longitudinal Study of Ageing (KLoSA), conducted by the Korea Employment Information Service. Representative samples of individuals aged 45 and older in Korea were collected in KLoSA and surveyed biennially from 2006 to 2020. Among the population surveyed during that period, individuals who responded that they had been diagnosed with cancer were included as study subjects (*N* = 791). Subsequently, the subjects were excluded based on the following criteria: (i) Those who reported a cancer diagnosis either before 2005 (*n* = 170) or after 2018 (*n* = 54); (ii) Those surveyed only once (*n* = 34); (iii) Those aged older than 65 (*n* = 334). Exclusion criteria (i) and (ii) were applied because the incidence of depressive symptoms due to job changes could not be defined unless the variable had been estimated at least twice. Exclusion criteria (iii) was applied, because the individuals aged over 65 may be in retirement, and such cases could exhibit distinct characteristics in job changes. Following these steps, the final sample consisted of 199 subjects. The average follow-up duration was 8.69 years. Every single survey period of each participant was counted as an ‘observation’, resulting in a total of 799 observations. These observations were categorized into four groups according to changes in work status: continuous unemployment, getting a job, quitting a job, and continuous employment (Fig. 1).

### Measures

The dataset was generated using 12 variables, which included changes in work status as the exposure variable, depressive symptoms as the outcome variable, and ten covariates.

### Exposure variable

The exposure variables, changes in work status are described in Additional file 1, in detail. The job status of participants was assessed by questionnaires each survey year and the change of the status compared to the previous survey year was converted into a ‘changes in work status’. Therefore, the exposure groups were four in total: i) Continuous unemployment (unemployment → unemployment), ii) Getting a job (unemployment → employment), iii) Quitting a job (employment → unemployment), and iv) Continuous employment (employment → employment). In other words, getting a job refers to a case where a participant who reported being unemployed in the previous survey had secured a job by the time of the follow-up survey. Similarly, quitting a job means a situation in which a participant who had a job at the time of the previous survey lost his or her job from then until the time of the follow-up survey.

### Outcome variable

Getting depressive symptoms is defined by CES-D-10 (Center for Epidemiological Studies-Depression Scale 10). It was screened in the general population, not diagnosed by a psychiatrist’s diagnosis, so we expressed it as depressive symptoms instead of depression [18]. KLoSA used the Korean version of the CES-D-10 [19] and different forms during the follow-up survey period. From the first to fourth survey years, the Andersen Form [20] was used and the Boston Form [21] has been in use since the fifth survey year. The Andersen and Boston forms are 10-item short versions based on the original CES-D-20 scale, with three items being different between the two forms. Andersen form consists of two factors–positive and negative affect–, and Boston form is composed of four factors–depressed affect, positive affect, somatic complaints, and interpersonal problems. Although there are differences between the two versions, they are both consistent with CES-D-20 [20, 21]. Each survey item was rated on a 4-point Likert scale and the overall score ranges from 0 to 30, indicating a higher score for more severe depressive symptoms. We adopted cut-off scores of 10 because of its good sensitivity and specificity [22, 23]. The occurrence of depressive symptoms was checked at each observation, and when the event was detected, the investigation ended (Additional file 1).

### Covariates

The covariates included the number of household members, education level, sex, age, marital status, occupations, cancer treatment, cancer type, catastrophic healthcare expenditure (CHE), and survivorship.

The number of household members was classified into two categories: multi-person and single-person households. The level of education was categorized into two groups: Above high school education or below. Marital status was categorized as ‘married’ and ‘single’, with the single group including separated, divorced, and widowed individuals. Occupations were categorized into four groups: (i) unemployed; (ii) managers, professionals, and clerks; (iii) service and sales workers; and (iv) craft and elementary workers. Craft and elementary workers consisted of (1) skilled agricultural, forestry, and fishery workers, (2) craft and related trades workers, (3) plant and machine operators, and assemblers, and (4) elementary occupations (e.g., cleaning and building-related occupations, and deliverers).

In addition, the four characteristics related to cancer were defined as follows. The cancer treatment variable was based on whether symptomatic relief therapy or chemotherapy was administered. Cancer types were categorized into two groups based on their tendency for developing depressive symptoms. Oropharyngeal, pancreatic, breast, and lung cancer have a high prevalence of depression, and colon cancer, gynecological cancer, and lymphoma have a low prevalence [14]. In this dataset, liver, gastric, pulmonary, and breast cancer were classified into the ‘high tendency to get depressive symptoms’ group, and colorectal, thyroid, cervical, ovary, prostate cancer, and others were categorized into the ‘low tendency to get depressive symptoms’ group. Catastrophic healthcare expenditure was defined as healthcare spending exceeding 20% of household income. The cut-off for CHE can vary depending on the healthcare situation in each country [24] and this study referenced the research [25] that used the same South Korean data to examine the association with cancer. Survivorship refers to the follow-up time after cancer diagnosis. We checked the linear-shaped distribution of survivorship and used it as a continuous variable to offer more information than a categorized one. There were 259 missing values in healthcare expenditure, 3 in household income, 1 in cancer types, and 11 in occupations and these were imputed using MissForest [26].

### Statistical analysis

In this study, the odds ratio (OR) was estimated using multivariable Generalized Estimating Equation (GEE) with an exchangeable working correlation structure, to investigate the association between changes in work status and depressive symptoms. Additionally, sampling weights were utilized to calculate the estimates at the population level and to reduce the occurrence of selection bias generated from sampling [27–29]. We further explored the subgroup analysis and multiplicative interaction between changes in work status and cancer types on depressive symptoms. In addition, we conducted a sensitivity analysis to confirm the robustness of the main results by cut-off scores of 10 on depressive symptoms, comparing the cut-off point of 4 when the overall score was calculated from 0 to 10–commonly used in the Korean version of CES-D-10 [30, 31].

All statistical tests were evaluated using a two-tailed 95% confidence interval (CI), and *P* < 0.05 was considered statistically significant. All statistical analyses were performed using R version 4.2.3.

## Results

Table 1 shows the distribution of employment-related variables and incidence of depressive symptoms by changes in work status. The total number of participants (N) was 199, with 799 observations. The number of observations with continued unemployment was 448, while continued employment was observed in 245 cases. There were 58 cases in which participants quit a job, and 48 cases involved getting a job.

In the continuous unemployment group, 54.1% of participants had depressive symptoms, while in the continuous employment group, 34.5% of individuals had depressive symptoms. The incidence of depressive symptoms was 33.3% in the group who quit their job.

The majority of participants who continued unemployment were unemployed at the time of cancer diagnosis (73.4%). Among the participants who quit their jobs, service and sales workers accounted for 60.0%. In the group who secured their job after a cancer diagnosis, 41.4% of workers were engaged in craft and elementary occupations. The distribution of socio-demographic factors was similar overall between the four groups (Additional file 2).

**Table 1:** Baseline characteristics of participants (*N* = 199, observations = 799)

Variables		Changes in work status				*P* value ^a^
		Continuous unemployment (*n* = 109)	Getting a job (*n* = 17)	Quitting a job (*n* = 15)	Continuous employment (*n* = 58)	
Depressive symptoms, n (%)		59 (54.1)	8 (47.1)	5 (33.3)	20 (34.5)	0.072
Job status& occupation, n (%)	Unemployed	80 (73.4)	12 (70.6)	1 (6.7)	5 (8.6)	< 0.001
	Manager, Professionals & Clerks	10 (9.2)	1 (5.9)	1 (6.7)	15 (25.9)	
	Service & sales workers	4 (3.7)	0 (0.0)	9 (60.0)	14 (24.1)	
	Craft & elementary workers	15 (13.8)	4 (23.5)	4 (26.7)	24 (41.4)	
Total observations		448	48	58	245	

^a^*P* values were obtained from the Fisher exact test

**Fig. 2:**
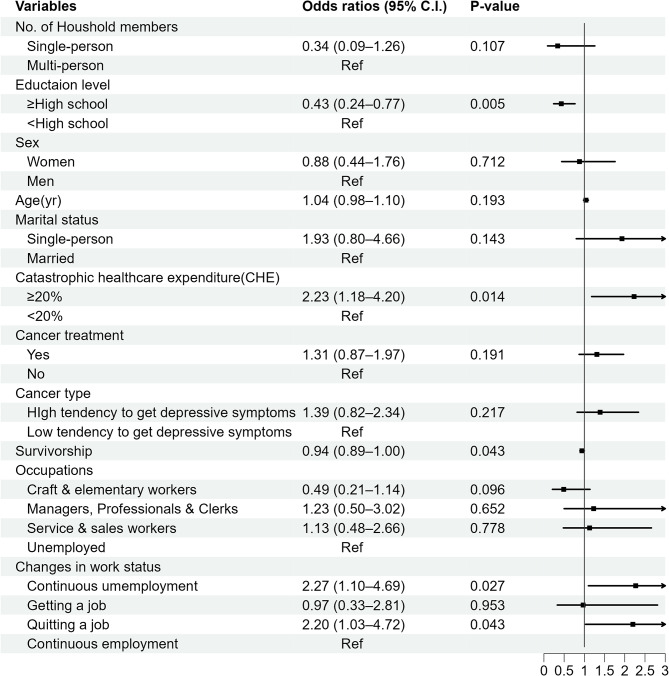
The OR of depressive symptoms with a cut-off point of 10 (*N* = 199, Observations = 799). † Adjusted for the number of household members, education level, sex, age, marital status, CHE, cancer treatment, cancer type, survivorship, and occupations

Figure 2 shows the odds ratio of depressive symptoms calculated from the GEE method and 95% confidence interval. Survivorship, the time since being diagnosed with cancer was related to the depressive symptoms. As the time increased in the one-year unit, the odds of depressive symptoms decreased (OR = 0.94, 95% CI = 0.89–1.00, *P* = 0.043). Changes in work status were associated with depressive symptoms in most groups. The odds of the continuous unemployment group were 2.27 times higher compared to the odds of the continuous employment group (OR = 2.27, 95% CI = 1.10–4.69, *P* = 0.027). The odds of the group quitting a job were 2.20 times higher than the odds of the continuous employment group (OR = 2.20, 95% CI = 1.03–4.72, *P* = 0.043). Getting a job, however, was not associated with getting depressive symptoms. Moreover, the number of household members, sex, age, marital status, cancer treatment, cancer type, and occupations also had no associations with depressive symptoms, except for education level (OR = 0.43, 95% CI = 0.24–0.77, *P* = 0.005). Catastrophic healthcare expenditure was associated with depressive symptoms. CHE more than 20% increased the odds of depressive symptoms by 2.23 times (OR = 2.23, 95% CI = 1.18–4.20, *P* = 0.014).

In the subgroup analysis (Table 2), continued unemployment was associated with depressive symptoms in cancer types with a low tendency to depression (OR = 3.19, 95% CI = 1.12–9.06). For the subgroup with cancer types of a high tendency to depression, however, any changes in work status had no association with depressive symptoms, and there was also no statistically significant interaction between cancer types and changes in work status on depressive symptoms (P for interaction > 0.05).

**Table 2:** Association between changes in work status and depressive symptoms by cancer types

Changes in work status	Cancer types		*P* for interaction ^b^
	A low tendency to depression ^a^ (*N* = 112, Observations = 447)	A high tendency to depression ^a^ (*N* = 87, Observations = 352)	
Continuous unemployment	3.19 (1.12–9.06)	1.48 (0.48–4.57)	0.686
Getting a job	0.98 (0.15–6.48)	0.92 (0.22–3.89)	0.958
Quitting a job	2.16 (0.88–5.28)	2.29 (0.68–7.74)	0.606
Continuous employment	1.00 [Ref.]	1.00 [Ref.]	-

^a^ Odds ratio (95% C.I.)

^a^ Adjusted for the number of household members, education level, sex, age, marital status, CHE, cancer treatment, cancer type, survivorship, and occupations

^b^ Interaction model of changes in work status with cancer types on depressive symptoms was adjusted for covariates as listed above

**Fig. 3:**
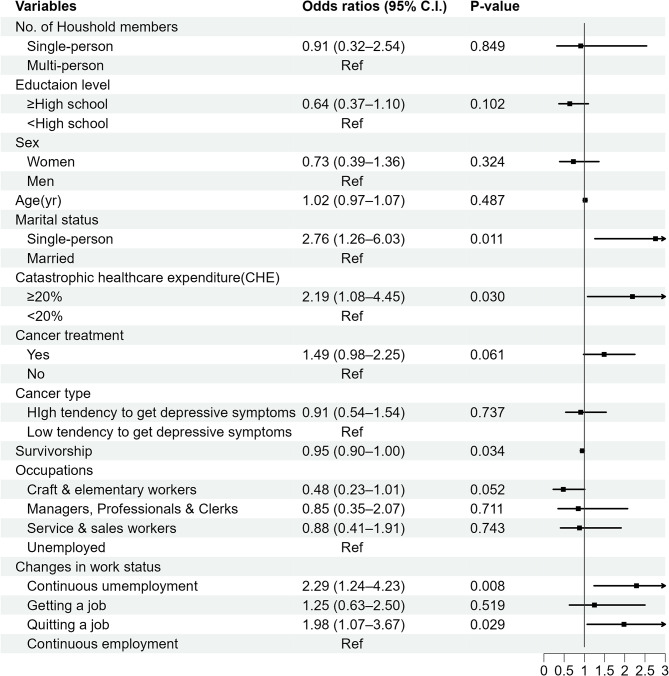
The OR of depressive symptoms with a cut-off point of 4 (*N* = 199, Observations = 799). † Adjusted for the number of household members, education level, sex, age, marital status, CHE, cancer treatment, cancer type, survivorship, and occupations

Figure 3 presents the result of the sensitivity analysis. When defining depressive symptoms as a cut-off point of 4 or higher, similar results were obtained compared to the main analysis where the cut-off point was defined as 10 or higher. The odds of the continuous unemployment group were 2.29 times higher compared to the odds of the continuous employment group (OR = 2.29, 95% CI = 1.24–4.23, *P* = 0.008). The odds of the group quitting a job were 1.98 times higher than the odds of the continuous employment group (OR = 1.98, 95% CI = 1.07–3.67, *P* = 0.029). In addition, as survivorship increased in a one-year unit, the odds of depressive symptoms decreased (OR = 0.95, 95% CI = 0.90–1.00, *P* = 0.034). CHE more than 20% increased the odds of depressive symptoms by 2.19 times (OR = 2.19, 95% CI = 1.08–4.45, *P* = 0.030).

## Discussion

This study identified an association between changes in work status and depressive symptoms among cancer survivors in South Korea. The main finding indicated that unemployment was associated with depressive symptoms among cancer survivors. Cancer survivors who quit a job and those who continued unemployment status were more likely to experience depressive symptoms. The early stage of survivorship was also associated with depressive symptoms. In other words, individuals who were recently diagnosed with cancer were more likely to experience depressive symptoms.

Cancer can have a negative impact on all aspects of a person’s well-being including physical, psychological, social, and mental health. Individuals with cancer that cannot work anymore, have difficulties sustaining a spiritual bond with the community or a sense of normalcy are at risk for low resilience [10, 32]. A lack of resilience, resulting from continued unemployment or job loss, is a psychosocial adversity and might lead to depressive symptoms for cancer survivors [33, 34]. It is widely known that stress can arise from work [35], however, our results were consistent with previous studies showing that cancer survivors tend to feel healthier when they are working [17, 36]. In this study, it was also noticeable that a number of subjects discontinued their jobs or maintained the discontinuation after quitting a job. Even though more workers maintained their jobs after a cancer diagnosis in this study, similar to prior research by Mols, et al. [37], caution is needed in interpreting it as many of employed cancer survivors tend to continue their work despite insufficient job performance and/or discomfort generated from cancer [12]. Empirical investigations accentuate the indispensable role of resilience in cancer survivors for both their mental and physical health [38, 39].

Therefore, providing assistance for cancer survivors to continue their work or return to work may be essential and useful, enhancing resilience and lowering the risk of depressive symptoms. To maintain employment for cancer survivors, it can be helpful for employers to adjust workload and working hours, or temporarily switch their position to part-time [40] based on their condition. Continuing work may be facilitated by giving time to adapt to the challenges posed by cancer and balance their work accordingly [32]. For cancer survivors who have stopped working but want to return to work, connecting them with specialists who can assist with returning to work or find alternative employment may be benefical. These can be ensured under labor protection policies and a supportive work culture for cancer survivors [32].

Cancer survivors in the acute survival stage can also be easily depressed because they face shocking words and have more unstable conditions that require severe treatment [41]. After escaping this phase, the depressive symptoms can be subsided gradually following their alleviated condition and adaption to the environment, but cannot be excluded that there was resilience rebounding on the adversities. It was a similar result to the previous study reporting that depressive symptoms tend to be at the peak with cancer diagnosis and thereafter start to decrease and be stable [42]. Proceeding from what has been said above, psychological care for cancer survivors may be crucial, particularly post or under primary cancer treatment period. Cancer survivors’ clubs or peer support groups may act as a place for sharing strategies to overcome challenges from those who have already proceeded through the recovery stage.

Catastrophic healthcare expenditure was associated with depressive symptoms. In South Korea, enrollment health insurance is mandatory and there have been several policies that limit out-of-pocket expenses for cancer patients to 5-10% [43]. Despite various financial support efforts, however, there is criticism that the current health security system has little impact on reducing the burden of medical expenses [44]. When cancer occurs, the burden of healthcare expenses is expanded [44, 45], and losing a job can be a double burden. Therefore, our results imply that thorough institutional review and improvement may be needed.

Meanwhile, cancer treatment was not associated with depressive symptoms, contrary to previous studies [46]. In the subgroup with cancer types of a low tendency to depression, continued unemployment was associated with depressive symptoms, while the subgroup with cancer types of a high tendency to depression had no association. There was no interaction between cancer types and changes in work status on depressive symptoms. For the low tendency cancers–colon cancer, gynecological cancer, and lymphoma–unemployment had a stronger explanatory power for depressive symptoms than other risk factors.

Socioeconomic status (SES) including the number of household members, marital status, and occupations had no association with depressive symptoms. In a previous study, living alone or unmarried status are high-risk factors of depression in the cancer journey [46], contrary to our study. High education level had lower odds of depressive symptoms, and this result was consistent with previous research showing that low education level had higher odds of depression [47].

In a sensitivity analysis using a different cut-off score of CES-D-10, both continued unemployment and quitting a job were still associated with depressive symptoms, and the beta estimates did not change considerably showing the robustness of the study results.

### Study limitations

Limitations in this study need to be considered. First, we were not able to include indicators of cancer severity, such as cancer stage, due to the absence of data, although changes in work status and depressive symptoms could be influenced by cancer severity [48, 49]. Second, despite the utilization of a large-scale dataset, the number of observations in each group was relatively small. It was also difficult to further subdivide cancer type, and we categorized it into two groups by depression tendencies. However, even with the wide confidence interval, statistically significant associations were identified at the 0.05 significance level. Third, we used integrated data measuring depressive symptoms by different versions: Andersen and Boston forms. Even though each version was confirmed to be consistent with the original CES-D-20, caution is needed when interpreting the results, as mentioned in previous research [10]. Last but not least, an in-depth study is needed to explore the specific intrinsic causes, such as low sense of coherence, optimism, hope, and societal elements, that hinder the formation of resilience or contribute to depressive symptoms after resignation from a job, to give shape to assistance for cancer survivors without a job.

### Study implications

It has been reported that, in the general population, job loss and depression—or depressive symptoms—have an association. Research has been needed to investigate the relationship among cancer survivors, as depression in cancer survivors is more critical to death or suicide than in the general population. Previous studies, however, have typically focused solely on either employment status or depression in cancer survivors. We bridged the research gap, exploring the association between different types of changes in work status and depressive symptoms among cancer survivors. Moreover, to date, although the effort to investigate employment issues among cancer survivors has arisen, progress has been hindered by the lack of longitudinal data and small sample size [50]. To dealt with this problem, we used 13 years of large longitudinal data across South Korea, and employed the GEE method and sampling weights.

## Conclusion

Cancer survivors who quit a job or continued unemployment were more likely to experience depressive symptoms and the early stage of survivorship was associated with depressive symptoms. These findings imply that assistance for continuing work or preparing alternative employment for cancer survivors may enhance resilience, lowering the risk of depressive symptoms. In addition, psychological care for cancer survivors may be crucial, particularly in the acute survival stage. Furthermore, financial support may be needed to reduce the burden of medical expenses for cancer survivors, as catastrophic healthcare expenditure can be a treat for depressive symptoms.

## Electronic supplementary material

Below is the link to the electronic supplementary material.

Supplementary Material 1

Supplementary Material 2

## Data Availability

The datasets analysed in this study are publicly available in the Korean Longitudinal Study of Ageing at https://survey.keis.or.kr/klosa/klosa04.jsp.

## Abbreviations

CES-D-10: Center for Epidemiological Studies-Depression Scale 10
CI: Confidence interval
GEE: Generalized estimating equation
IRB: Institutional Review Board
KLoSA: Korean Longitudinal Study of Ageing
OR: Odds ratio
CHE: Catastrophic healthcare expenditure
APA: American Psychological Association
