# Supplementary material for: Changes in work status after cancer diagnosis and their associations with depressive symptoms among cancer survivors: findings from the Korean longitudinal study of ageing
Source: BMC Psychol. 2024 Oct 14;12:551. doi: 10.1186/s40359-024-01970-9 (PMC11475630; doi:10.1186/s40359-024-01970-9)
Supplement: Supplementary file 1 — Supplementary Material 1 [file 40359_2024_1970_MOESM1_ESM.pdf]

Additional file 1

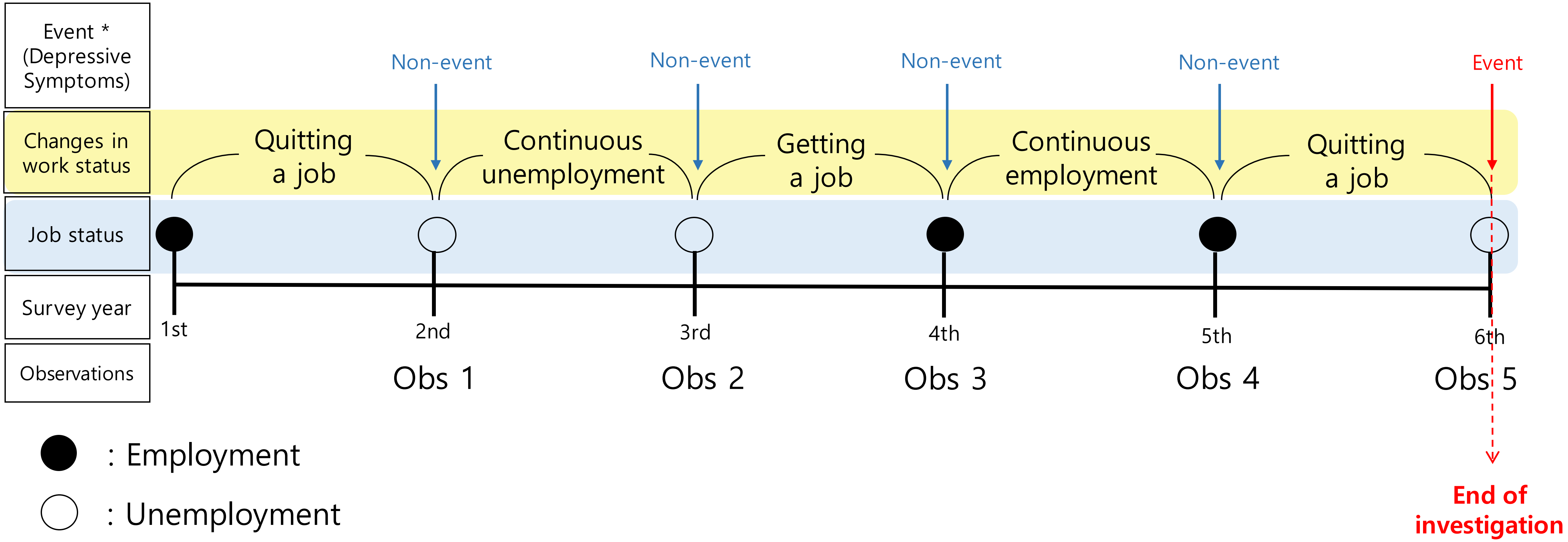

The definition of changes in work status for one hypothetical participant; an event case without loss to follow-up
