# Supplementary material for: Changes in work status after cancer diagnosis and their associations with depressive symptoms among cancer survivors: findings from the Korean longitudinal study of ageing
Source: BMC Psychol. 2024 Oct 14;12:551. doi: 10.1186/s40359-024-01970-9 (PMC11475630; doi:10.1186/s40359-024-01970-9)
Supplement: Supplementary file 2 — Supplementary Material 2 [file 40359_2024_1970_MOESM2_ESM.docx]

**Additional file 2** Baseline characteristics of participants (N=199, Observations= 799)

| Variables | | Changes in work ^b^ | | | | P-value ^a^ |
| --- | --- | --- | --- | --- | --- | --- |
|  |  | Continuous unemployment  (n = 109) | Getting a job  (n = 17) | Quitting a job  (n = 15) | Continuous employment  (n = 58) |  |
| No. of household members | Multi-person | 103 (94.5) | 17 (100.0) | 15 (100.0) | 54 (93.1) | 0.820 |
|  | Single-person | 6 ( 5.5) | 0 ( 0.0) | 0 (0.0) | 4 (6.9) |  |
| Education level | ≥High school | 52 (47.7) | 9 (52.9) | 8 (53.3) | 38 (65.5) | 0.180 |
|  | <High school | 57 (52.3) | 8 (47.1) | 7 (46.7) | 20 (34.5) |  |
| Sex | Women | 78 (71.6) | 9 (52.9) | 7 (46.7) | 28 (48.3) | 0.011* |
|  | Men | 31 (28.4) | 8 (47.1) | 8 (53.3) | 30 (51.7) |  |
| Age(yr) | | 58.3 (4.8) | 59.1 (5.2) | 58.1 (5.9) | 56.7 (4.9) | 0.154 |
| Marital status | Single | 14 (12.8) | 3 (17.6) | 0 (0.0) | 7 (12.1) | 0.486 |
|  | Married | 95 (87.2) | 14 (82.4) | 15 (100.0) | 51 (87.9) |  |
| Catastrophic healthcare expenditure  (CHE) | $\geq$ 20% | 12 (11.0) | 1 (5.9) | 4 (26.7) | 4 (6.9) | 0.165 |
|  | < 20% | 97 (89.0) | 16 (94.1) | 11 (73.3) | 54 (93.1) |  |
| Cancer treatment | Yes | 51 (46.8) | 7 (41.2) | 5 (33.3) | 18 (31.0) | 0.245 |
|  | No | 58 (53.2) | 10 (58.8) | 10 (66.7) | 40 (69.0) |  |
| Cancer type | High tendency to get depressive symptoms | 47 (43.1) | 9 (52.9) | 4 (26.7) | 27 (46.6) | 0.461 |
|  | Low tendency to get depressive symptoms | 62 (56.9) | 8 (47.1) | 11 (73.3) | 31 (53.4) |  |
| Survivorship (yr) | | 2.5 (1.0) | 2.4 (0.6) | 2.6 (0.6) | 2.5 (0.6) | 0.461 |
| Total observations | | 448 | 48 | 58 | 245 |  |

^a^ Global P values were obtained from fisher exact tests for categorical variables and F tests for continuous variables.

^b^ Values are mean (SD) for continuous variables and number (%) for categorical variables.

*p<.05
